# Characterization of volatile constituents and odorous compounds in peach (*Prunus persica L*) fruits of different varieties by gas chromatography–ion mobility spectrometry, gas chromatography–mass spectrometry, and relative odor activity value

**DOI:** 10.3389/fnut.2022.965796

**Published:** 2022-08-15

**Authors:** Ping Sun, Bing Xu, Yi Wang, Xianrui Lin, Chenfei Chen, Jianxi Zhu, Huijuan Jia, Xinwei Wang, Jiansheng Shen, Tao Feng

**Affiliations:** ^1^Jinhua Academy of Agricultural Sciences (Zhejiang Institute of Agricultural Machinery), Jinhua, China; ^2^School of Perfume and Aroma Technology, Shanghai Institute of Technology, Shanghai, China; ^3^The College of Agriculture and Biotechnology, Zhejiang University, Hangzhou, China; ^4^Zhengzhou Fruit Research Institute, Chinese Academy of Agricultural Sciences, Zhengzhou, China

**Keywords:** gas chromatography–ion mobility spectrometry (GC-IMS), peach, odorous compounds, gas chromatography–mass spectrometry (GC-MS), relative odor activity value (ROAV)

## Abstract

The aim of this study is to acquire information for future breeding efforts aimed at improving fruit quality *via* effects on aroma by comparing the diversity of Chinese local peach cultivars across 10 samples of three varieties (honey peach, yellow peach, and flat peach). The volatile components of peach fruits were analyzed and identified by gas chromatography–ion mobility spectrometry (GC-IMS) combined with gas chromatography–mass spectrometry (GC-MS), and the main flavor components of peach fruit were determined by relative odor activity value (ROAV) and principal component analysis (PCA). A total number of 57 volatile components were detected by GC-IMS, including eight aldehydes, nine alcohols, eight ketones, 22 esters, two acids, two phenols, two pyrazines, one thiophene, one benzene, and two furans. The proportion of esters was up to 38.6%. A total of 88 volatile components were detected by GC-MS, among which 40 were key aroma compounds, with an ROAV ≥ 1. The analysis results showed that alcohols, ketones, esters, and aldehydes contributed the most to the aroma of peach fruit. PCA demonstrated that (E,E)-2, 6-non-adienal, γ-decalactone, β-ionone, and hexyl hexanoate were the key contributors to the fruit aroma. A reference for future directional cultivation and breeding could be provided by this study through evaluating the aroma quality of the peach at the cultivar level. The possible reasonable application of these peach fruits pulp will be guided through these research.

## Introduction

Peach (*Prunus persica L.*) is a small deciduous tree with edible fruit belonging to the Rosaceae genus *Amycdalus L*. (1). The flowers can be ornamental, the fruit is juicy and can be eaten raw or canned, etc., and the kernels can also be eaten. Peaches with white and yellow flesh are referred to as “longevity peaches” and “immortal peaches.” It is also known as the “first fruit in the world” because of its delicious pulp (2). Its history dates back 4000 years. It originated in China and is widely cultivated in the latitude range of 23 to 45° north (3). Thanks to a long planting history and a vast planting area, diligent and intelligent laborers of China have cultivated a variety of colorful peach trees almost everywhere from the southern provinces of Jiangsu and Zhejiang to the northern province of Jilin (4). According to statistics, China is the origin of thousands of peach tree varieties (5).

Due to their thick, juicy flesh, rich aroma and high nutritional value, peaches are a popular fruit on the market (6). Currently, most research on the aroma of peach fruit focuses on the analysis and identification of aroma components of a single variety. However, there are few studies evaluating the aroma quality of peach fruit of different varieties. Therefore, it is necessary to establish and implement an objective method and system for evaluating the aroma of peach fruit. Currently, gas chromatography–ion migration spectrometry, headspace solid-phase microextraction–gas chromatography–mass spectrometry, gas chromatography–olfactometry, electronic nose, etc. are the primary analytical methods for volatile components of aroma (7).

Gas chromatography–ion mobility spectrometry (GC-IMS) is a detection technique that combines gas chromatography and ion mobility spectrometry. This technology overcomes the limitation of the poor separation degree in ion migration technology so that the ion migration signal response in gas chromatography can be identified by the difference in the ion migration rate in the electric field after gas-phase pre-separation, resulting in more chemical information from GC separation (8, 9). The technology is simple and sensitive and requires no pretreatment. It has a wide range of applications, including food flavor analysis and quality detection. Headspace solid-phase microextraction is a common method for the extraction of aroma substances from peach fruit, with the benefits of easy operation, less loss of aroma components, and high sensitivity (10, 11). The purpose of principle component analysis (PCA) is to recombine multiple indicators into several new comprehensive indicators by reducing the data dimensions in order to conduct a comprehensive evaluation (12). This method has been applied to evaluate the aroma quality of Dongbei suancai (13), Tricholoma matsutake Singer (14), and soybean whey tofu (15).

Therefore, in this study, GC-IMS technology was combined with HS-SPME-GC-MS technology and the PCA method to detect and analyze volatile substances in 10 kinds of peach samples of three varieties. Meanwhile, the ROAV method was used to identify key flavor substances in peach fruits. Numerous studies, such as Keemun black tea (16), orange juice (17), and faba bean (18) studies, had used this method for their aroma quality evaluation. In order to provide a theoretical basis for peach fruit variety differentiation, peach fruit aroma detection, and quality evaluation, the principal component comprehensive score model was developed to analyze the similarities and differences of aroma of 10 peach samples using the principal component analysis method. In addition, this study can aid in the selection of peach varieties suitable for public taste and breeding efforts aimed at genetically enhancing peach flavor.

## Materials and methods

### Experimental materials and reagents

The volatile compounds of honey peach (“Yuanmeng,” “Yihe,” “Yuandong,” “Baifeng,” “Wanhujing”), yellow peach (“Huangguifei,” “Jinxiu,” “Jinyuan”), and flat peach (“Ruipan-19,” “Yulu”) were studied. Because early August is the best maturation period for peach, we collected the samples from a local commercial orchard in Jinhua city, Zhejiang Province, on 1 August 2021. At maturity, ripe fruits free of physical damage and fungal infection were selected based on color, firmness, and aroma of the peaches by the local farmer. Ripe peaches were brought back to the laboratory in Shanghai on the same day, pureed, sealed, and stored in a –18°C freezer for analysis (within 24 h). A homologous series of alkanes (C_8_–C_30_) were purchased from Sigma Aldrich (St. Louis, MO, United States). Purified water was obtained using a Milli-Q purification system (Model Milli-Q Advantage A10, Millipore, Bedford, MA, United States).

### Methods

#### Gas chromatography sample pretreatment

In order to ensure the reliability of the carboxen polydimethyl siloxane (CAR-PDMS) fiber, it should be aged for 15 min at 250°C. In tandem with the description by Zhu and Xiao (4) and many pre-experiments, the best operational procedure was selected as follows: 5 g peach puree taken in 20-mL screw capped vials fitted with PTEE silicone septa. Then, 75 μm carboxen polydimethyl siloxane (CAR-PDMS) fiber was exposed to the headspace of the sample in a water bath at 60°C for 30 min. Thereafter, SPME fiber was directly introduced into the GC injector for desorption and analysis at 250°C for 5 min. All the experiments were performed in triplicate.

#### Conditions of gas chromatography–mass spectrometry

The samples were analyzed using the HP-5MS column (30 m × 0.25 mm × 0.25 μm, Agilent, Santa Clara, CA, United States), with purified helium as the carrier gas, at a constant flow rate of 1 ml/min. The oven temperature was maintained at 40°C for 5 min, then increased to 100°C at a rate of 3°C/min, increased to 230°C at a rate of 3°C/min, and finally held at 230°C for 5 min. The injector and FID temperatures were set to 250°C and 280°C, respectively. The injection port was set at 250°C with splitless mode. For mass spectrometry analysis, electron ionization (EI) at 70 eV was used, and the MS scanning was undertaken from 40 to 450 m/z. The ion source temperature was 230°C. The mass spectrometer was operated in the full scan mode, and ChemStation software (Agilent Technologies) was used to determine the area of each peak. Identification of volatile compounds was achieved by comparing the mass spectra with the data system library (NIST 20) and retention index. The experiments were performed in triplicate.

#### Conditions of gas chromatography–ion mobility spectrometry

Volatile fingerprint analysis of Jinhua peaches was performed using a GC-IMS composed of an Agilent 490 gas chromatograph (Agilent Technologies, United States) and an IMS instrument (FlavourSpec^®^, Gesellschaft für Analytische Sensorsysteme mbH, Dortmund, Germany). The GC apparatus was equipped with an autosampler (CTC Analytics AG, Zwingen, Switzerland) device that connected to a headspace sampling unit. Before GC-IMS analysis, each peach sample (5 mL) was transferred into the headspace sampling vial (20 mL) and incubated at 40°C for 20 min of equilibration. Thereafter, 200 μL of headspace gas was extracted using a heated (50°C) syringe and automatically injected by the autosampler (in the splitless mode). For GC analysis, volatile compounds were separated by using an FS-SE-54-CB-1 capillary column (15 m × 0.53 mm, 1 μm film thickness) at 40°C with nitrogen (> 99.95% purity) used as carrier gas using the programmed procedure: 2 mL/min for 2 min, increased to 10 mL/min within 10 min, and further increased to 150 mL/min within 10 min. The IMS instrument was programmed at 45°C with a constant drift gas (nitrogen, > 99.95% purity) flow in a drift tube under a flow rate of 150 mL/min. The retention index (RI) of each compound was calculated using n-ketones C_4_–C_12_ (Sinopharm Chemical Reagent Beijing Co., Ltd., China) as external references (19).

### Qualitative and quantitative analyses of aroma components

#### Data processing of the gas chromatography–mass spectrometry

Matching retention times of authentic standards, retention indices (RIs), and mass spectra in the NIST20 database identified the compounds. RIs were determined by injecting the homologous series of alkanes (C_8_ - C_30_) (6). The retention index was calculated as shown in Equation 1:


(1) Retentionindex=100n+100(t-at)n/(t-n+1t)n

In the formula, t_a_ is the retention time of the chromatographic peak a and t_n_,t_n+1_ is the retention time of C*n*_n_ and C_n+1_ in orthoalkanes.

The relative content of each volatile compound in peach fruit was calculated by peak area normalization:


(2) Relativeamount(%)=M/N×100

In the formula, M is the peak area of individual component aroma substances and N is the overall peak area.

#### Data processing of the gas chromatography–ion mobility spectrometry

The qualitative analysis of volatile compounds was determined by the RI value and drift time of standards in the GC-IMS library (Gesellschaft für Analytische Sensorsysteme mbH, Dortmund, Germany). The quantitative analysis of volatile compounds was determined by the signal intensity of each compound obtained using Laboratory Analytical Viewer (LAV, G.A.S., Dortmund, Germany). In addition, the fingerprints and the difference profiles of volatile molecules in peaches were obtained by Reporter and Gallery plug-ins. All analyses were performed in triplicate.

### Determination of main flavor compounds in peach fruit by the relative odor activity value method

With reference to the ROAV method, the contribution of volatile flavor substances in peach fruit was evaluated, and then the main flavor substances were determined. The threshold in ROAV calculation was selected by olfactory thresholds in water taken from the literature (20). Compounds with ROAVs equal to or greater than 1 were actually the main flavor components of the analyzed samples, and compounds with ROAVs greater than 0.1 and less than 1 play a embellish role in aroma (21). The components that contribute most to the total flavor of a solid peach sample were defined as follows:

ROAV_max_ = 100, then other components (a):


(3) ROAV≈a100×C/aC×maxT/maxTa

In the formula, C_a_ is the relative content of the volatile components (%), T_a_ is the sensory threshold for the volatile components (ppb), and C_max_,T_max_ is the relative contents of volatile components with the largest contribution to the total aroma of samples (%) and their sensory threshold (ppb).

### Statistical analysis

GC × IMS Library Search V 2.2.1 analysis software, built-in NIST database, and IMS database were used for qualitative analysis of volatile flavor substances in the samples. The Reporter, Gallery Plot, and Dynamic PCA plug-ins were combined with Laboratory Analytical Viewer (LAV, G.A.S., Dortmund, Germany) to establish standard curves of samples for quantitative analysis, and then the differences of volatile organic compounds among samples of honey peach, yellow peach, and flat peach were compared intuitively. The data from GC-MS were standardized using TBtools (Toolbox for Biologists) v1.098696 software for heat map, and principal components analysis (PCA) was used to evaluate similarity and difference using Origin 2022 software.

## Results and discussion

### Fingerprints of volatile compounds in peaches by HS-gas chromatography–ion mobility spectrometry analysis

**FIGURE 1:**
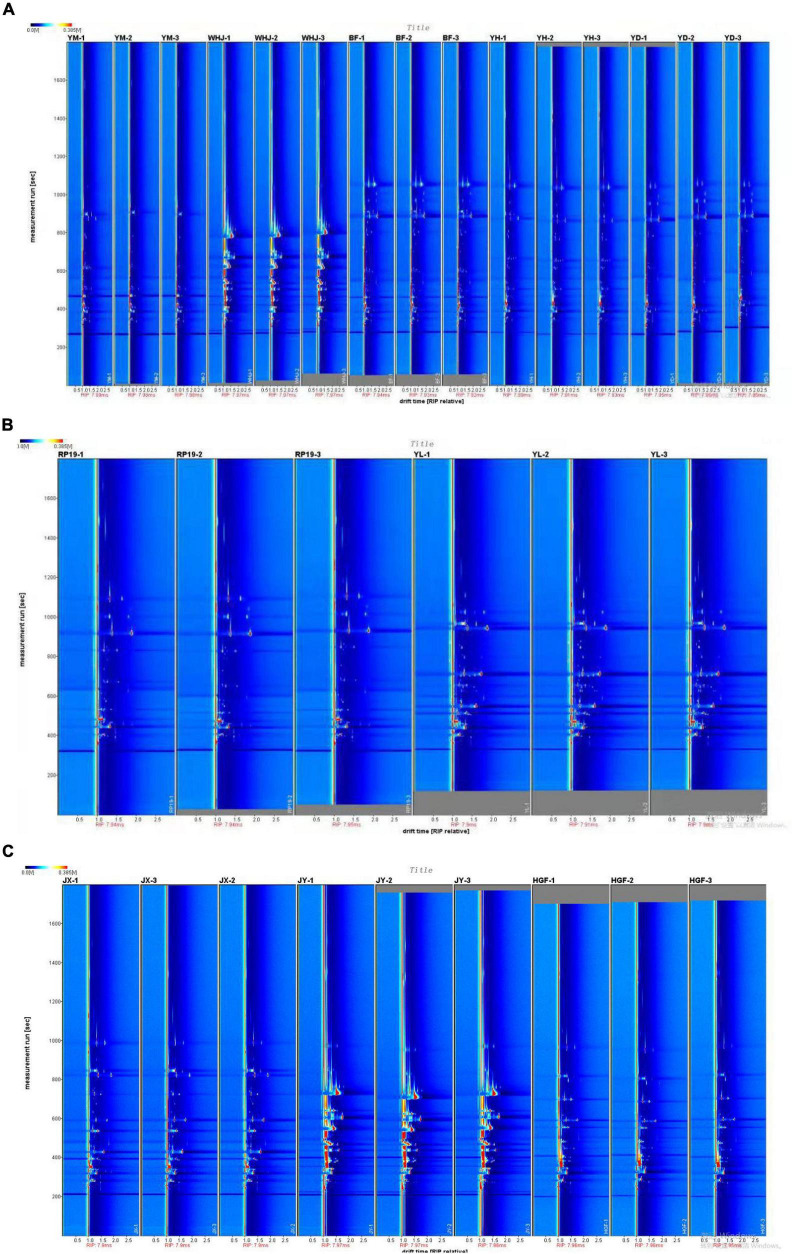
Gas-phase ion migration spectrum of peach samples [**(A)**-honey peach, **(B)**-flat peach, **(C)**-yellow peach].

The background of the two-dimensional top view generated by the Reporter plug-in is blue, and the vertical red line at abscissa 1.0 represents the RIP peak (reaction ion peak, normalized). According to the presence or shade of a peak (color point), differences in composition and concentration between different samples can be visualized. The vertical axis represents the gas chromatography retention time (s), while the horizontal axis represents the ion migration time (normalized treatment). Each point on either side of the RIP peak represents a volatile organic compound. The color represents the concentration of the substance; white indicates a low concentration, and red indicates a high concentration; the darker the color, the higher the concentration. Difffferentiation spectrum is a method to analyze the difference of GC-IMS spectrum (top view). It uses a particular sample as a reference and compares all volatile substances in different samples to determine their differences. Red indicates that the concentration of volatile substances in the sample is greater than that in the reference sample, whereas blue indicates that the concentration of volatile substances in the sample is less than that in the reference sample. Figure 1A demonstrates that the volatile organic compounds of “Baifeng,” “Yihe,” and “Yuandong” peaches were similar, whereas the volatile organic compounds of “Yuanmeng” and “Wanhujing” peaches were vastly dissimilar from the other three peach samples. Figure 1C shows that the volatile organic compounds of “Jinxiu” and “Huangguifei” yellow peaches were similar, whereas the differences between “Jinyuan” and the other two samples were obvious. Figure 1B found that the volatile organic compounds of “Ruipan- 19” and “Yulu” flat peaches were similar, but the volatile compounds of “Yulu” were demonstrably greater than those of “Ruipan-19”.

In HS-GC-IMS, volatile compounds with varying retention times were ionized separately by the ion source to form molecular ion groups (15). Table 1 presents the volatile component information obtained *via* GC-IMS analysis. By comparing the relative retention index (RI) and the drift time (Dt) of the standard in the GC-IMS library, volatile compounds were identified (22). As shown in Table 1, the GC-IMS profiles of the 10 samples revealed that the C chains of 57 volatile organic compounds were all within the range of C_4_ ∼ C_12_; these included eight aldehydes, nine alcohols, eight ketones, 22 esters, two acids, two phenols, two pyrazines, one thiophene, one benzene, and two furans. The majority of the 57 known volatile components qualitatively identified were prevalent aroma components in peach fruit. Rarely reported in the study of peach fruit volatile components were neryl acetate, citronellyl acetate, geranyl formate, bread thiophene, methyl ortho-anisate, 2-ethylpyrazine, 2-pentylfuran, diethyl malonate, cumin aldehyde, and other volatile components. These substances typically had unique aromas, such as neryl acetate, which had a rose-like odor; citronellyl acetate, which had pear and apple aromas; diethyl malonate, which had apple and pineapple aromas; bread thiophene, which had sweet, almond, fruity, heliotrope, and nutty flavors; and 2-ethylpyrazine, which smelled like nuts, fish, meat, potatoes, and coco.

**TABLE 1:** Qualitative results for all volatile components on the basis of gas chromatography–ion mobility spectrometry (GC-IMS).

No.	Name	CAS	Molecular formula	Molecular weight	RI^a^			RT^b^(s)			DT^c^(ms)			Aroma description
					Honey	Flat	Yellow	Honey	Flat	Yellow	Honey	Flat	Yellow	
1	p-Anisaldehyde	123-11-5	C_8_H_8_O_2_	136.15	1268.2	—	—	898.55994	—	—	1.231	—	—	sweet floral balsam
2	Phenylacetaldehyde	122-78-1	C_8_H_8_O	120.15	1054.1	—	—	614.445	—	—	1.554	—	—	green sweet floral
3	Tetrahydrothiophene	1003-04-9	C_4_H_6_OS	102.15	809.5	—	—	387.65997	—	—	1.327	—	—	garlic meaty green
4	allyl isothiocyanate	57-06-7	C_4_H_5_NS	99.15	918.2	912	—	472.68	467.41498	—	1.11	1.395	—	strong pungent mustard
5	*cis*-dihydrocarvone	3792-53-8	C_10_H_16_O	152.23344	1183	—	—	772.98	—	—	1.313	—	—	herbal warm
6	Isophorone	78-59-1	C_9_H_14_O	138.21	1132.5	—	—	711.94495	—	—	1.263	—	—	woody sweet green
7	2-non-anone	821-55-6	C_9_H_18_O	142.24	1122.5	—	—	700.44	—	—	1.4	—	—	cheesy green fruity,
8	isoamyl butyrate	106-27-4	C_9_H_18_O_2_	158.24	1091.4	—	—	663.975	—	—	1.401	—	—	fruity green apricot
9	2-Octanol	123-96-6	C_8_H_18_O	130.23	1005.6	—	—	555.555	—	—	1.446	—	—	fresh spicy green
10	ethyl 3-methylbutyrate	108-64-5	C_7_H_14_O_2_	130.18	885.6	—	—	445.37997	—	—	1.255	—	—	fruity sweet apple
11	2-Methylbutanal	96-17-3	C_5_H_10_O	86.13	729.8	—	—	335.205	—	—	1.16	—	—	musty cocoa phenolic
12	4-ketoisophorone	1125-21-9	C_9_H_12_O_2_	152.19	1108.7	—	—	684.83997	—	—	1.326	—	—	citrus floral musty
13	Hexan-2-one	591-78-6	C_6_H_12_O	100.16	848.6	—	—	416.32498	—	—	1.202	—	—	fruity fungal meaty buttery
14	geranyl formate	105-86-2	C_11_H_18_O_2_	182.26	1326.3	—	—	1006.98	—	—	1.853	—	—	green floral citrus
15	Methyl anisate	121-98-2	C_9_H_10_O_3_	166.17	1308.5	1334.5	—	970.12494	1024.5299	—	1.808	1.814	—	herbal anise sweet
16	Neryl acetate	141-12-8	C_12_H_20_O_2_	196.29	1380.6	—	—	1128.6599	—	—	1.239	—	—	floral rose soapy citrus
17	citronellyl acetate	150-84-5	C_12_H_22_O_2_	198.3	1408.8	—	—	1197.2999	—	—	1.482	—	—	floral green rose fruity
18	Isobornyl acetate	125-12-2	C_12_H_20_O_2_	196.29	1294.2	—	—	941.45996	—	—	1.385	—	—	herbal woody sweet
19	Isopulegyl acetate	89-49-6	C_12_H_20_O_2_	196.286	—	1290.4	—	—	934.82996	—	—	1.386	—	minty leafy
20	2-Hexen-1-ol	2305-21-7	C_6_H_12_O	100.16	—	916.8	—	—	471.50998	—	—	1.183	—	fruity green leafy
21	Isopentyl formate	110-45-2	C_6_H_12_O_2_	116.16	—	824.7	830.1	—	398.58	402.47998	—	1.277	1.267	fruit green
22	2-Methyl-1-butanol	137-32-6	C_5_H_12_O	88.15	824.7	817.5	829.3	398.58	393.31497	401.895	1.24	1.471	1.47	ethereal fusel alcoholic fatty
23	Aniline	62-53-3	C_6_H_7_N	93.13	—	959.3	—	—	509.53497	—	—	1.423	—	unknown
24	2-formyl-5-methylthiophene	13679-70-4	C_6_H_6_OS	126.18	1065.7	1086	1156	629.45996	656.565	739.6395	1.169	1.173	1.176	sweet almond cherry
25	Methyl benzoate	93-58-3	C_8_H_8_O_2_	136.15	—	1105.9	—	—	681.72	—	—	1.206	—	wintergreen almond floral
26	Ethyl benzoate	93-89-0	C_9_H_10_O_2_	150.17	1218.1	1209	—	821.73	808.47	—	1.248	1.262	1218.1	fruity dry musty sweet
27	p-Methyl guaiacol	93-51-6	C_8_H_10_O_2_	138.16	1214.3	1230.2	—	816.07495	839.67	—	1.179	1.179	1214.3	spicy clove woody leathery
28	Methyl 2-methoxybenzoate	606-45-1	C_9_H_10_O_3_	166.17	—	1307.2	—	—	967.39496	—	—	1.234	—	herbal floral fruity
29	Geranyl acetate	105-87-3	C_12_H_20_O_2_	196.29	—	1427.7	—	—	1245.855	—	—	1.224	—	floral rose lavender green
30	Thymol	89-83-8	C_10_H_14_O	150.22	1241.8	1255.7	—	857.22	878.67	—	1.253	1.25	—	herbal thyme phenolic
31	2-Methylpropanal	78-84-2	C_4_H_8_O	72.11	—	—	635.1	—	—	281.97	—	—	1.112	fresh aldehydic floral green
32	2-Pentanone	107-87-9	C_5_H_10_O	86.13	—	—	761.7	—	—	355.28998	—	—	1.137	sweet fruity ethereal
33	Pentanal	110-62-3	C_5_H_10_O	86.13	756.9	—	735.6	352.16998	—	338.715	1.187	—	1.171	bready fruity nutty berry
34	Isopentanal	590-86-3	C_5_H_10_O	86.13	—	—	734.6	—	—	338.12997	—	—	1.395	chocolate peach fatty
35	3-Methyl-2-butenal	107-86-8	C_5_H_8_O	84.12	845.8	—	819.6	414.18	—	394.875	1.088	—	1.364	sweet fruity pungent
36	Butyl acetate	123-86-4	C_6_H_12_O_2_	116.16	—	—	865	—	—	428.99997	—	—	1.609	fruity sweet banana
37	2-Methylbutanol	616-16-0	C_5_H_12_O	88.15	—	—	809	—	—	387.27	—	—	1.473	fermented fatty
38	Methional	3268-49-3	C_4_H_8_OS	104.17	—	—	988.7	—	—	537.615	—	—	1.391	musty potato tomato
39	Methyl 2-furoate	611-13-2	C_6_H_6_O_3_	126.11	—	956.4	991.9	—	506.805	540.735	—	1.477	1.171	fruity mushroom sweet
40	Ethyl 2-hydroxy-4-methylpentanoate	10348-47-7	C_8_H_16_O_3_	160.2108	—	—	1037	—	—	592.995	—	—	1.299	fresh black berry^d^
41	Octen-3-ol	3391-86-4	C_8_H_16_O	128.21	—	—	1035.4	—	—	591.045	—	—	1.738	mushroom earthy green
42	2-formyl-5-methylthiophene	13925-00-3	C_6_H_8_N_2_	108.14	—	—	1068.5	—	—	633.165	—	—	1.179	peanut butter musty
43	3-isopropyl-2-methoxypyrazine	25773-40-4	C_8_H_12_N_2_O	152.19	—	—	1143.4	—	—	724.62	—	—	1.24	pea earthy beany
44	Cumin aldehyde	122-03-2	C_10_H_12_O	148.20	—	—	1219.1	—	—	823.095	—	—	1.891	spicy cumin green herbal
45	p-Cymen-7-ol	536-60-7	C_10_H_14_O	150.22	1265.2	1307.8	1334	893.685	968.75995	1023.36	1.887	1.33	1.326	harsh plastic acrylate fruity
46	Methyl chavicol	140-67-0	C_10_H_12_O	148.2	1198.4	—	1234.7	793.25995	—	846.3	1.239	—	1.231	sweet spice green
47	ethyl acrylate	140-88-5	C_5_H_8_O_2_	100.12	—	—	744.9	—	—	344.565	—	—	1.401	harsh plastic acrylate fruity
48	2-pentyl furan	3777-69-3	C_9_H_14_O	138.21	—	—	1025.8	—	—	579.345	—	—	1.252	fruity green earthy
49	3-Methyl valeric acid	105-43-1	C_6_H_12_O_2_	116.16	955.5	—	994.4	506.025	—	543.26996	1.605	—	1.253	cheesy green fruity sweaty
50	diethyl malonate	105-53-3	C_7_H_12_O_4_	160.17	1079.7	—	1078.9	647.985	—	647.00995	1.252	—	1.257	sweet fruity green apple
51	Propyl butanoate	557-00-6	C_8_H_16_O_2_	144.21	—	—	929.1	—	—	482.235	—	—	1.252	bitter sweet apple fruity
52	2-Butylfuran	4466-24-4	C_8_H_12_O	124.18	—	—	932	—	—	484.77	—	—	1.191	fruity wine sweet spicy
53	3-Octanol	589-98-0	C_8_H_18_O	130.2279	—	—	1005.6	—	—	555.555	—	—	1.385	earthy mushroom herbal melon
54	1-Phenylethyl acetate	93-92-5	C_10_H_12_O_2_	164.2	—	—	1218.1	—	—	821.73	—	—	1.067	fruity berry green
55	Benzyl acetate	140-11-4	C_9_H_10_O_2_	150.17	1197.5	1201.8	1216.7	792.08997	798.13495	819.58496	1.313	1.312	1.323	sweet floral fruity jasmin
56	Isopulegol	89-79-2	C_10_H_18_O	154.2493	—	—	1191.7	—	—	783.89996	—	—	1.381	minty cooling medicinal woody
57	6-Methyl-5-hepten-2-one	110-93-0	C_8_H_14_O	126.2	—	—	1008.2	—	—	558.48	—	—	1.168	citrus green musty lemongrass

^a^ RI, retention index;^b^ RT, retention time;^c^ DT, migration time; ^d^ G Lytra, S Tempere, G De Revel, JC Lytra et al. (23); “–” indicates not detected.

**FIGURE 2:**
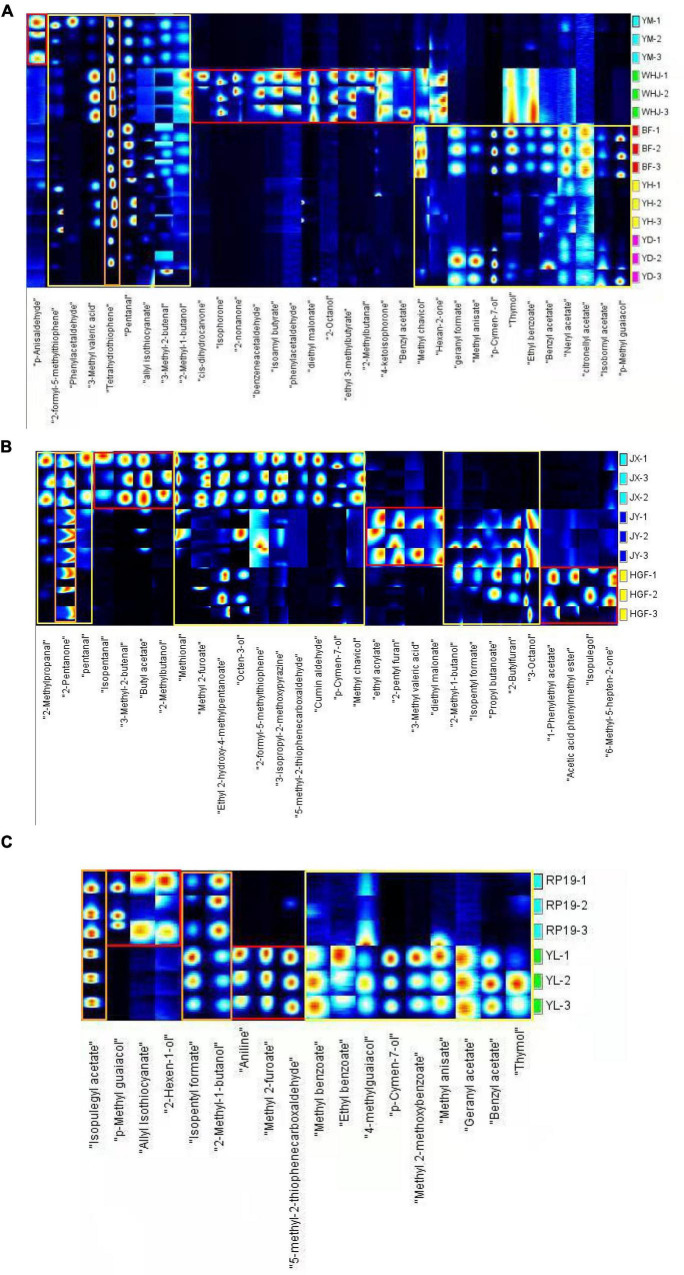
Gallery Plot of volatile organic compounds in peach samples [**(A)**-honey peach; **(B)**-yellow peach; **(C)**-flat peach].

Figure 2 depicts the volatile organic compound (VOC) fingerprints of various peaches, including honey peaches, yellow peaches, and flat peaches. Each row represents the signal peaks extracted from a single sample, while each column represents the signal peaks of the same volatile organic compounds extracted from multiple samples. Figure 2 also shows the complete VOC information of each sample and the differences between the peach samples. The fingerprint spectrum of Gallery Plot revealed the variation of flavoring substances in various samples more clearly. Each column represents a flavor substance in various samples, with the color intensity representing the concentration level. Through longitudinal comparison, the concentration of various flavoring substances revealed a very intuitive rule. The fingerprints of volatile organic compounds collected from peach fruits were roughly divided into three distinct color regions, with the red region indicating that the volatile organic compounds in this region were unique to certain peach fruits, or that their concentration was significantly higher than that in other regions. The yellow area indicates that the volatile organic compound content of various peach fruits varied greatly in this region, whereas the orange area indicates that there were less differences between large peach samples in this region. Figure 2A demonstrates that “Yuanmeng,” “Wanhujing,” “Baifeng,” “Yihe,” and “Yuandong” peaches had a high concentration of 3-tetrahydrothiophenone and possessed special aromas such as green vegetables and butter. A total of five honey peach samples had significantly different levels of volatile organic compounds, namely, 3-methylpentanoic acid, valeraldehyde, allyl isothiocyanate, 3-methyl-2-butenal, and 2-methyl-1-butanol, and the odor of these compounds was predominantly fruity. “Wanhujing” and “Yihe” contained significantly more 3-methyl-pentanoic acid than the other three varieties. “Baifeng” and “Yihe” contained more valeraldehyde, while “Yuandong” and “Baifeng” contained more geranyl formate. Anisaldehyde was a special flavoring component in “Yuanmeng,” whereas cis-dihydrocarvone, isophorone, 2-non-one, and diethyl malonate were special ingredients in “Wanhujing”. Figure 2B demonstrates that the 2-pentanone content of “Jinxiu,” “Jinyuan,” and “Huangguifei” yellow peaches were relatively high, exhibiting a sweet fruit and banana flavor with a slight variation in fermentation. The distinctive components of “Jinxiu” were iso-valeraldehyde, 3-methyl-2-butenal, and (R)-2-methyl-1-butanol. Specific volatile organic compounds of “Jinyuan” were ethyl acrylate, 2-pentylfuran, 3-methylpentanoic acid, and diethyl malonate. The distinctive constituents of “Huangguifei” included sulfenyl acetate, benzyl acetate, methyl heptenone, and isopropyl alcohol. Jinxiu contained higher levels of methyl 2-furoate, cumin aldehyde, and pyrazine than “Jinyuan” and “Huangguifei,” while “Huangguifei” contained more 3-octanol. As shown in Figure 2C, the content levels of iso-humenthol acetate, iso-amyl formate, and 2-methyl butanol in “Ruipan-19” and “Yulu” flat peach were high, indicating the flavor of fruit and fragrance. The specific volatile organic compounds of Ruipan-19 included 4-methylguaiacol, 2-hexenol, and allyl isothiocyanate, whereas “Yulu” had aminobenzene, bread thiophene, and methyl 2-furoate. “Yulu” contained more volatile organic compounds, such as methyl benzoate, benzyl acetate, and geranyl acetate, than “Ruipan-19.”

### Distinction of different peach samples by principal component analysis

**FIGURE 3:**
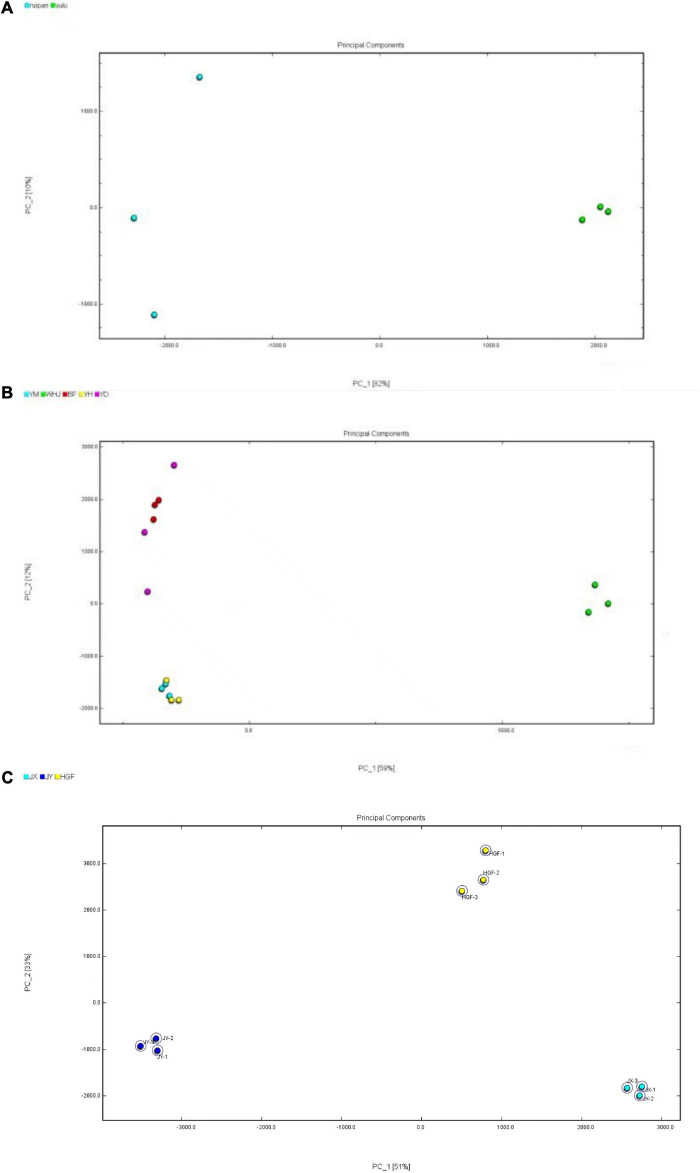
Principal component analysis (PCA) diagrams of peach samples [**(A)**-honey peach; **(B)**-yellow peach; **(C)**-flat peach].

Based on volatile components of peach fruits identified through GC-IMS, PCA statistics were used to analyze different honey peaches (such as the “Yuanmeng,” “Wanhujing,” “Baifeng”, “Yihe” and “Yuandong”), yellow peaches (such as “Jinxiu,” “Jinyuan,” “Huangguifei”), and flat peaches (such as “Ruipan-19” and “Yulu”). Figure 3 depicts the results of Dynamic PCA plug-in analysis. Figure 3A demonstrates that PC-1 and PC-2 had variance contribution rates of 59% and 12%, respectively, and a cumulative variance contribution of 71%, which is greater than the trusted value of 60%. Consequently, PC-1 and PC-2 are adequate to reflect the distinctions between various peach samples. In the PCA diagram, a close distance between samples indicates a small difference, whereas a far distance indicates a significant difference. As shown in Figure 3A, the differences between the three parallel groups of the same sample were negligible, whereas the volatile organic compounds in the peach fruits of different peaches were significantly diverse. The “Wanhujing” peach, which was the most special among the five kinds of honey peaches, was distinct from the other four varieties. The close distance between “Yuanmeng” and “Yihe” honey peaches indicated that their volatile composition and flavor were similar. Figure 3B reveals that the cumulative variance contribution rate of PC-1 and PC-2 was 88%, which can reflect the difference between the three yellow peach samples. It was evident from that the distance between different kinds of yellow peaches was quite large, indicating that the similarity of volatile organic compounds in these three yellow peach varieties was low. Similarly, Figure 3C demonstrates that the composition of volatile organic compounds in “Ruipan-19” and “Yulu” flat peach was relatively not identical. Consequently, GC-IMS results combined with PCA can quickly and easily differentiate between various peach samples. In addition, this method can be served as a guide for distinguishing peach fruit from different producing regions, harvesting stages, and storage methods.

### Gas chromatography–mass spectrometry analysis of peach fruit in different peach varieties

Names and relative contents of volatile flavor compounds in peach fruits of different varieties are shown in Table 2. A total of 88 kinds of volatile flavor compounds were detected in 10 peaches. The quantity and relative content of volatile flavor compounds in peach samples were different.

**TABLE 2:** Relative content of volatile compounds in peach samples based on gas chromatography–mass spectrometry (GC-MS).

No.	Name	CAS	Aroma description	Relative content (%)									
				Honey peach					Yellow peach			Flat peach	
				YM	YD	YH	WHJ	BF	HGF	JX	JY	RP19	YL
1	benzaldehyde	100-52-7	sweet bitter almond cherry	2.288	1.988	0.622	0.800	1.044	—	0.369	0.069	0.140	0.629
2	(E)-2-hexenal	6728-26-3	green banana fatty cheesy	0.751	1.206	1.383	0.622	0.544	—	0.080	0.018	0.092	0.851
3	benzoic acid	65-85-0	faint balsam urine	0.531	0.370	—	—	—	—	—	—	—	—
4	para-cymene	99-87-6	fresh citrus terpene woody	0.390	0.101	—	—	—	0.443	—	—	—	—
5	(E,E)-2,4-heptadienal	4313-03-5	fatty green oily	0.385	0.167	—	—	—	—	—	—	—	—
6	4-carvomenthenol	562-74-3	pepper woody earth musty sweet	0.378	—	—	—	—	—	—	—	—	—
7	camphene	79-92-5	woody herbal fir	0.238	—	—	0.093	—	—	—	—	—	—
8	isoquinoline	119-65-3	sweet balsam herbal	0.237	—	—	—	—	—	—	—	—	—
9	(E)-2-octenal	2548-87-0	fresh cucumber fatty green	0.210	—	—	—	—	—	—	—	—	—
10	cyclohexanol	108-93-0	camphor menthol phenol	0.194	—	—	2.020	—	—	—	—	—	—
11	γ-hexalactone	695-06-7	herbal coconut sweet creamy	0.177	—	—	—	—	—	—	—	—	—
12	non-anal	124-19-6	waxy aldehydic rose fresh	0.152	—	—	0.055	—	—	—	—	—	—
13	styrene	100-42-5	sweet balsam floral	0.141	0.091	0.100	—	—	—	0.063	—	—	—
14	geranyl acetone	689-67-8	floral fruity rose	0.130	—	—	—	—	—	—	—	—	—
15	dihydro-β-ionol	3293-47-8	woody floral amber	0.114	—	—	—	0.245	—	—	—	—	—
16	acetophenone	98-86-2	sweet pungent hawthorn	0.112	—	—	—	—	—	—	0.039	—	—
17	ethyl palmitate	628-97-7	waxy fruity creamy	0.111	—	—	—	—	—	—	—	—	—
18	3,5-octadien-2-one	38284-27-4	fruity fatty mushroom	0.100	—	—	—	—	—	—	—	—	—
19	meta-tolualdehyde	620-23-5	sweet fruity cherry	0.084	—	—	—	—	—	—	0.000	—	—
20	(E,E)-2,6-non-adienal	17587-33-6	fresh citrus green	0.083	—	—	—	—	—	—	—	—	—
21	cedrol	77-53-2	woody amber floral sweet	0.073	—	—	—	0.828	—	—	—	—	—
22	phenol	108-95-2	phenolic plastic rubber	0.072	—	—	—	1.505	—	—	—	—	—
23	2-ethyl-1-hexanol	104-76-7	sweet fatty fruity	0.068	—	—	—	—	—	—	—	—	—
24	δ-tridecalactone	7370-92-5	creamy coconut dairy	0.028	—	—	—	—	—	—	—	—	—
25	para-anisyl nitrile	874-90-8	sweet floral hawthorn	0.028	—	—	—	—	—	—	—	—	—
26	γ-decalactone	706-14-9	fruity creamy peach	—	2.111	—	1.359	2.936	—	—	—	—	—
27	(E)-geranyl acetone	3796-70-1	floral fruity green	—	1.526	0.382	0.556	—	—	—	—	0.104	—
28	β-ionone	14901-07-6	floral woody sweet fruity	—	1.310	—	0.474	—	—	—	—	—	—
29	hexanol	111-27-3	green fruity sweet	—	1.152	—	3.304	1.173	—	—	—	—	—
30	2-octanone	111-13-7	earthy weedy natural woody herbal	—	0.702	—	—	—	—	—	—	—	—
31	amber naphthofuran	3738-00-9	dry woody amber sweet	—	0.672	—	—	—	—	—	—	—	—
32	(E,E)-farnesyl acetate	4128-17-0	oily waxy	—	0.351	—	—	—	—	—	—	—	—
33	dextro-limonene	5989-27-5	citrus orange fresh sweet	—	0.277	0.184	0.070	—	—	0.087	—	—	—
34	γ-octalactone	104-50-7	sweet coconut peach	—	0.235	—	—	—	—	—	—	—	—
35	ipsdienol	35628-00-3	pine balsamic	—	0.218	—	0.237	—	—	—	—	—	—
36	β-cyclocitral	432-25-7	tropical saffron herbal fruity	—	0.206	—	—	—	—	—	—	—	—
37	linalool	78-70-6	citrus floral sweet rose	—	0.200	—	—	—	—	—	—	—	—
38	(E)-2-hexen-1-yl hexanoate	53398-86-0	green natural cognac herbal	—	0.195	—	0.038	—	—	—	—	—	—
39	(E)-2-tridecenal	7069-41-2	waxy fatty citrus creamy	—	0.132	—	—	—	—	—	—	—	—
40	(E)-3-hexen-1-ol	928-97-2	fruity green cortex floral	—	0.131	—	0.243	—	—	—	—	—	—
41	myrcene	123-35-3	peppery terpene spicy	—	0.128	—	—	—	0.289	—	—	—	—
42	hexyl hexanoate	6378-65-0	herbal fresh sweet fruity	—	0.114	0.314	0.122	—	—	—	—	0.064	0.525
43	β-ocimene	13877-91-3	citrus tropical green	—	0.101	—	—	—	—	—	—	—	—
44	dihydro-β-ionone	17283-81-7	earthy woody mahogany	—	0.067	0.240	1.488	—	—	—	—	0.068	0.328
45	non-adecane	629-92-5	bland	—	0.056	—	—	—	—	—	—	—	—
46	anethol	104-46-1	sweet anise licorice	—	0.049	—	—	—	—	—	—	—	—
47	bornyl acetate	76-49-3	woody pine herbal	—	0.049	—	0.091	—	—	—	—	—	—
48	cis-allocimene	7216-56-0	grass floral	—	0.034	—	—	—	—	—	—	—	—
49	γ-dodecalactone	2305-05-7	fatty peach sweet fruity	—	—	2.500	—	—	0.130	—	—	—	—
50	(E)-2-hexenoic acid	13419-69-7	fruity sweet	—	—	1.297	—	—	—	—	—	—	2.251
51	hexanoic acid	142-62-1	sour fatty sweat cheese	—	—	0.399	—	—	—	—	—	—	—
52	(E)-β-ionone	79-77-6	powdery floral woody berry	—	—	0.293	—	—	—	1.428	—	—	—
53	hexanal	66-25-1	fresh green fatty fruity	—	—	0.224	—	0.068	—	—	—	—	0.258
54	(E)-4-hexen-1-ol	928-92-7	green herbal musty	—	—	0.194	—	—	—	—	—	—	—
55	Costol	515-20-8	—	—	—	—	1.952	—	—	—	—	—	—
56	alloaromadendrene	25246-27-9	woody	—	—	—	0.874	—	—	—	0.397	—	—
57	eugenyl acetate	93-28-7	fresh sweet woody	—	—	—	0.840	—	—	—	—	—	—
58	(-)-spathulenol	77171-55-2	honey	—	—	—	0.739	—	—	—	—	—	—
59	β-irone	79-70-9	fresh sweet violet fruity	—	—	—	0.595	—	—	—	—	—	—
60	theaspirane	36431-72-8	tea herbal green	—	—	—	0.249	—	—	—	—	—	—
61	beta-santalol	77-42-9	woody	—	—	—	0.196	—	—	—	—	—	—
62	valencene	4630-07-3	sweet fresh citrus grapefruit	—	—	—	0.195	—	—	—	—	—	—
63	ethyl benzene	100-41-4	—	—	—	—	0.147	—	—	—	—	—	—
64	hexyl acetate	142-92-7	fruity green apple banana	—	—	—	0.127	—	—	—	—	0.026	—
65	3,7-dimethyl-1-octene	4984-01-4	woody, piney, herbaceous	—	—	—	0.126	—	—	—	—	—	—
66	hexahydrofarnesyl acetone	502-69-2	oily sweet green melon	—	—	—	0.039	—	—	—	—	—	—
67	β-caryophyllene	87-44-5	sweet woody spice	—	—	—	—	0.904	—	—	—	—	—
68	(E)-2-hexen-1-ol	928-95-0	fresh green leafy fruity	—	—	—	—	0.229	—	—	—	—	—
69	(Z)-3-hexen-1-ol	928-96-1	fresh green cut grass	—	—	—	—	0.138	—	—	—	—	—
70	isocaryophyllene	118-65-0	woody spicy	—	—	—	—	0.061	—	—	—	—	—
71	α-guaiene	3691-12-1	sweet woody balsam peppery	—	—	—	—	0.033	—	—	—	—	—
72	meta-dimethyl hydroquinone	151-10-0	acid fruity nutmeg neroli	—	—	—	—	0.025	—	—	—	—	—
73	α-pinene	80-56-8	fresh camphor sweet	—	—	—	—	—	3.827	—	—	—	—
74	(E)-β-ocimene	3779-61-1	sweet herbal	—	—	—	—	—	0.267	—	—	—	—
75	para-methyl acetophenone	122-00-9	sweet creamy fruity	—	—	—	—	—	0.163	—	—	—	—
76	2-pentyl furan	3777-69-3	fruity green earthy	—	—	—	—	—	0.088	—	—	—	—
77	tetradecane	629-59-4	mild waxy	—	—	—	—	—	—	0.045	—	—	—
78	ethyl valerate	539-82-2	sweet fruity apple	—	—	—	—	—	—	0.060	—	—	—
79	watermelon ketone	28940-11-6	fresh watery clean melon	—	—	—	—	—	—	0.662	—	—	—
80	(E,E)-2,4-hexadienal	142-83-6	sweet green spicy floral	—	—	—	—	—	—	—	0.296	—	—
81	dipentene	138-86-3	citrus herbal terpene	—	—	—	—	—	—	—	0.019	0.100	—
82	(+)-aromadendrene	489-39-4	wood	—	—	—	—	—	—	—	6.799	—	—
83	1-pentylpyrrole	699-22-9	green fatty	—	—	—	—	—	—	—	4.922	—	—
84	γ-Selinene	515-17-3	woody	—	—	—	—	—	—	—	16.38	—	—
85	Butyric acid	107-92-6	sour chessy fruity	—	—	—	—	—	—	—	—	0.048	—
86	Hexanoic acid,5-methyl-	628-46-6	fatty chessy oily fruity	—	—	—	—	—	—	—	—	0.128	—
87	Heptanoic acid	111-14-8	waxy chessy fruity	—	—	—	—	—	—	—	—	—	1.351
88	Eugenol	97-53-0	sweet spicy woody	—	—	—	—	—	—	—	—	—	1.152

“–” indicates not detected.

Esters are considered major contributors to fruity and floral aromas, and high levels of esters should give peaches a pleasant flavor (24). A total of eight esters were found in peach samples, namely, ethyl palmitate (17), (E,E)-farnesyl acetate (32), (E)-2-hexen-1-yl hexanoate (38), hexyl hexanoate (42), bornyl acetate (47), eugenyl acetate (57), hexyl acetate (64), and ethyl valerate (78), contributing to 9.09% of the total volatiles (Table 2). Among the three groups, the sum of the esters was highest in honey peach and lower in the yellow peach group. Moreover, a significantly higher content of total esters was found in “Wanhujing” and “Yuandong” than in “Yuanmeng,” “Yihe,” and “Baifeng” (Table 2). Regarding individual ester contents, the most abundant content of hexyl acetate (64) was found in “Wanhujing.” High hexyl hexanoate (42) levels were observed in “Yulu,” with contents of about 0.525% (Table 2). For the other six esters, the levels were generally lower than 0.1%; therefore, it is not meaningful to be discussed.

There were five lactones found in groups, namely, γ-hexalactone (11), δ-tridecalactone (24), γ-decalactone (26), γ-octalactone (34), and γ-dodecalactone (49), and these accounted for 5.68% of the total volatiles. The most abundant content of compounds is γ-decalactone (26) in honey peach of “Baifeng” and “Yuandong,” except “Yuanmeng” and “Wanhujing,” and high γ-dodecalactone (49) levels were observed in “Yihe.” Flat peach had not been detected any lactones. In yellow peach, “Huangguifei” had very high levels of γ-dodecalactone (49), with 0.13%. The other three lactones were found only in individual peaches, for example, γ-octalactone (34) was higher in “Yuandong,” with 0.235%. Lactones, particularly γ-decalactone and -decalactone, have been reported as “character-affecting” compounds in peach and nectarine aromas. In this study, honey peaches had significantly a higher content of γ-decalactone (26) than yellow peaches and flat peaches. However, Wang et al. (25) concluded that flat peaches contain significantly higher amounts of γ-decalactone (26) than other peaches. There was significant differences between honey peaches and other two peaches.

In total, 11 aldehydes were detected, and benzaldehyde (1) and (E)-2-hexenal (2) were dominant, with contents ranging from 0 to 2.288% and 0 to 1.206%, respectively (Table 2). “Yuanmeng” had the highest benzaldehyde content, with 2.288%, and “Yuandong” had the most abundant content of (E)-2-hexenal among all the taxa, with 1.206%. In all samples, only “Huangguifei” was not detected with aldehydes. Several taxa, “Yihe,” “Baifeng,” and “Yulu,” had high levels of hexanal (53), ranging from 0 to 0.258%. For the other eight aldehydes, the levels were generally lower, except “Yuanmeng” and “Yuandong,” where (E,E)-2,4-heptadienal (5) was abundant, with 0.385% and 0.167%, respectively; high β-cyclocitral (36) levels were observed in “Yuandong,” with 0.206%; and “Jinyuani” had very high levels of (E,E)-2,4-hexadienal (80), with 0.296%.

The total content of terpenoids accounted for 18.18% of total volatiles. The sum of terpenoids in “Yuandong” and “Wanhujing” was significantly higher than in other samples, and it was also significantly higher in yellow peaches. Of 16 terpenoids found, dextro-limonene (33) was the major compound. There was no terpenoid detected in “Yulu,” but dextro-limonene was high in some cultivars with honey peach and yellow peach: “Yuandong,” “Yihe,” “Wanhujing,” and “Jinxiu.” The second abundant terpenoid was styrene (13). Levels of dextro-limonene tended to be positively correlated with the styrene content, with high levels in “Yuandong,” “Yihe,” and “Jinxiu.” High (+)-aromadendrene (82) levels were observed in “Jinyuan,” with 6.799%. “Huangguifei” had the most abundant content of α-pinene (73), with 3.827%.

In this research, 12 ketones were found, including (E)-geranyl acetone (27), β-ionone (28), 2-octanone (30), dihydro-β-ionone (44), (E)-β-ionone (52), and watermelon ketone (79), contributing to 13.64% of the total volatiles. The sum of ketones in “Wanhujing” was significantly higher than that in “Baifeng,” “Huanguifei,” “Jinyuan,” and “Yulu.” Dihydro-β-ionone (44) and (E)-geranyl acetone (27) were dominant, with contents ranging from 0 to 1.488% and 0 to 1.526%, respectively (Table 3). “Wanhujing” had the most abundant content of dihydro-β-ionone among all the taxa (44), with 1.488%, and highest (E)-geranyl acetone (27) levels were observed in “Yuandong,” with contents of about 1.526%. The lowest dihydro-β-ionone (44) and (E)-geranyl acetone (27) levels were detected in “Ruipan-19,” with 0.104% and 0.068%, respectively. Almost all samples were detected with ketones, and the contents of ketones in peaches were relatively high, with about 0.1% or more, but there was no found in “Baifeng.”

**TABLE 3:** Volatile components of ROAV ≥ 1 in peach samples and their thresholds and aroma notes.

No.	Name	CAS	Threshold^a^(ppb)	ROAV										Note
				YM	YD	YH	WHJ	BF	HGF	JX	JY	RP19	YL	
1	Benzaldehyde	100-52-7	750.9	100.0	1.439	–	–	–	–	–	1.857	100.0	70.67	sweet
2	(E)-2-hexenal	6728-26-3	88.7	277.9	—	—	1.191	—	—	—	4.105	559.1	811.3	green
3	benzoic acid	65-85-0	568	30.68	—	—	—	—	—	—	—	—	—	fatty
4	para-cymene	99-87-6	5.01	2554	1.051	—	—	—	32.34	—	—	—	—	citrus
5	4-carvomenthenol	562-74-3	1200	10.34	—	—	—	—	—	—	—	—	—	spicy
6	(E)-2-octenal	2548-87-0	3	2300	—	—	—	—	—	—	—	—	—	green
7	cyclohexanol	108-93-0	470	13.52	—	—	—	—	—	—	—	—	—	green
8	γ-hexalactone	695-06-7	260	22.37	—	—	—	—	—	—	—	—	—	sweet
9	non-anal	124-19-6	1.1	1978	—	—	8.463	—	—	—	—	—	—	aldehydic
10	styrene	100-42-5	3.6	1281	1.310	—	—	—	—	—	—	—	—	sweet
11	geranyl acetone	689-67-8	60	71.17	—	—	—	—	—	—	—	—	—	floral
12	acetophenone	98-86-2	65	56.35	—	—	—	—	—	—	12.24	—	—	sweet
13	ethyl palmitate	628-97-7	2000	1.815	—	—	—	—	—	—	—	—	—	fruity
14	(E,E)-2,6-non-adienal	17587-33-6	0.5	5422	—	—	—	—	—	—	—	—	—	citrus
15	γ-decalactone	706-14-9	1.1	—	100.0	—	209.4	100	—	—	—	—	—	peach
16	(E)-geranyl acetone	3796-70-1	60	—	1.325	—	1.570	—	—	—	—	927.8	—	floral
17	β-ionone	14901-07-6	3.5	—	19.50	—	22.96	—	—	—	—	—	—	sweet
18	hexanol	111-27-3	5.6	—	10.72	—	100	7.850	—	—	—	—	—	fruity
19	γ-octalactone	104-50-7	12	—	1.018	—	—	—	—	—	—	—	—	peach
20	β-cyclocitral	432-25-7	5	—	2.148	—	—	—	—	—	—	—	—	fruity
21	linalool	78-70-6	0.22	—	47.40	—	—	—	—	—	—	—	—	green
22	myrcene	123-35-3	1.2	—	5.548	—	—	—	87.94	—	—	—	—	spicy
23	γ-dodecalactone	2305-05-7	0.43	—	—	100.0	—	—	110.1	—	—	—	—	peach
24	(E)-β-ionone	79-77-6	0.007	—	—	720.1	—	—	—	100.0	—	—	—	floral
25	dihydro-β-ionone	17283-81-7	3.6	—	—	1.148	70.05	—	—	—	—	10145	7685	woody
26	(Z)-3-hexen-1-ol	928-96-1	3.9	—	—	—	—	1.325	—	—	—	—	—	green
27	α-pinene	80-56-8	14	—	—	—	—	—	100.0	—	—	—	—	green
28	(E)-β-ocimene	3779-61-1	34	—	—	—	—	—	2.868	—	—	—	—	herbal
29	para-methyl acetophenone	122-00-9	21	—	—	—	—	—	2.834	—	—	—	—	sweet
30	2-pentyl furan	3777-69-3	5.8	—	—	—	—	—	5.518	—	—	—	—	fruity
31	(E,E)-2,4-hexadienal	142-83-6	60	—	—	—	—	—	—	—	100.0	—	—	green
32	dipentene	138-86-3	200	—	—	—	—	—	—	—	1.888	267.8	—	citrus
33	hexyl acetate	142-92-7	115	—	—	—	—	—	—	—	—	119.2	—	fruity
34	Butyric acid	107-92-6	2400	—	—	—	—	—	—	—	—	10.78	—	sour
35	hexyl hexanoate	6378-65-0	6400	—	—	—	—	—	—	—	—	5.345	6.926	fruity
36	Hexanoic acid,5-methyl-	628-46-6	4600	—	—	—	—	—	—	—	—	14.92	—	fatty
37	(E)-2-hexenoic acid	13419-69-7	1900	—	—	—	—	—	—	—	—	—	100.0	sour
38	Heptanoic acid	111-14-8	640	—	—	—	—	—	—	—	—	—	178.1	fruity
39	Eugenol	97-53-0	0.71	—	—	—	—	—	—	—	—	—	136930	spicy
40	hexanal	66-25-1	5	—	—	—	—	—	—	—	—	—	4358	fruity

^a^Olfactory thresholds in water taken from the literature (20). “–” indicates not detected.

A total of 15 alcohols were only found in honey peach, accounting for 17.05% of top compounds. Among them, “Wanhujing” contained the largest number of alcohols, while the content of each alcohol had high rates. High hexanol (29) levels were observed in “Jinyuan,” “Wanhujing,” and “Baifeng,” with contents of 1.152%, 3.304%, and 1.173%, respectively. As results showed, the difference between honey peaches and other kinds of peaches was definitely significant. A total of six acids were found in this work, namely, benzoic acid (2), (E)-2-hexenoic acid (50), hexanoic acid (51), butyric acid (85), hexanoic acid, 5-methyl- (86), and heptanoic acid (87). For the six acids, the levels were generally lower, except in “Yulu” where heptanoic acid (87) and (E)-2-hexenoic acid (50) were abundant, with 1.351% and 2.251%, respectively. Also high (E)-2-hexenoic acid (50) levels were detected in “Yihe,” with a content of 1.297%. In general, the content of acid substances in flat peaches was higher than that in honey peaches and yellow peaches.

Because of the number of rest compounds detected in peaches was much lower, we did not discuss in detail. But there was something interesting that we found: “Baifeng” and “Yulu” had high phenol levels, such as eugenol (88) and phenol (22), with contents of 1.505% and 1.152%, respectively, and 1-pentylpyrrole (83) and γ-selinene (84) were dominant, with contents of 4.922% and 16.389%, respectively, in “Jinyuan.”

In combination with Table 2, honey peach contained an abundance of volatile components, whereas volatile components of yellow peach and flat peach contained were relatively lower. The relative content of terpenes in all peach species was relatively lower, whereas the relative content of alcohols, esters, ketones, and aldehydes was significantly higher, and this result was generally consistent with the fingerprints of volatile components detected by GC-IMS (Figure 2). The relative content analysis suggested that alcohols, esters, and aldehydes were likely the primary sources of the distinctive aroma substances in peach fruit.

### Heat map analysis of the content of volatile components in peach fruit of different varieties

**FIGURE 4:**
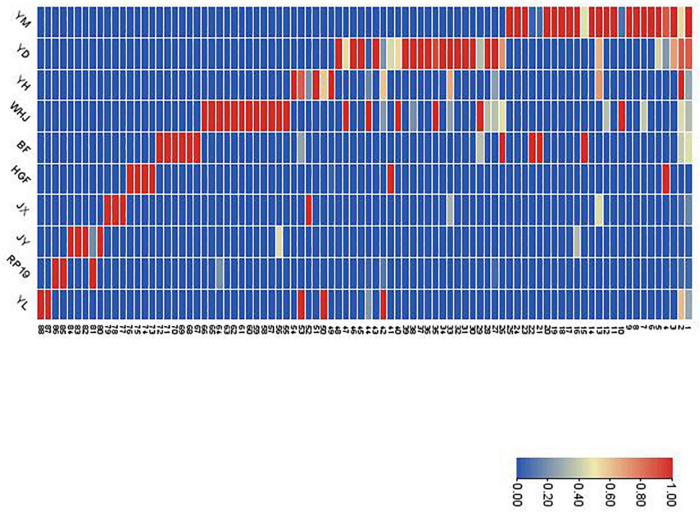
Relative contents (as shown by heat map) of all volatile compounds in the fruits of 10 peach samples. The codes in this figure correspond to the codes of cultivars in Table 2.

Figure 4 shows that the components and contents of volatile substances in 10 kinds of peach fruits were significantly different. According to the cluster analysis of volatile compounds in the heat map, except for “Huangguifei,” “Jinxiu,” “Jinyuan,” and “Ruipan-19,” the red and pink regions of volatile compounds in the heat map of other varieties were mainly benzaldehyde (1) and (E)-2-hexenal (2). The heat maps of volatile substances of “Jinyuan” and “Ruipan-19” were concentrated at limonene (81). The volatile substances of “Jinxiu,” “Yuandong,” and “Yihe” heat maps in the red region were concentrated in styrene (13). In addition, the red part of heat maps of “Yuandong,” “Wanhujing,” and “Baifeng” also concentrated on prodecalactone (26), while the red part of the heat maps of “Yulu” and “Yihe” concentrated in (E)-2-hexenoic acid (50) and hexanal (53).

### Relative odor activity value analysis of volatile flavor compounds in peach fruits of different varieties

A variety of volatile flavor compounds were detected in different varieties of peach fruit, but only some of them contributed to the overall flavor of peach fruit, and the rest only played a role in modifying and synergizing the flavor of peach fruit. The contribution of volatile flavor substances to the flavor characteristics of peach fruit is determined by their relative content and aroma threshold. The aroma threshold is a minimum olfactory value of odor. Therefore, the relative content of flavor substances cannot explain their contribution to the flavor of peach fruit. As a result, ROAV analysis was carried out based on the threshold value of each flavor substance. The compounds with an ROAV ≥ 1 had a greater contribution to the aroma of the sample and were the key aroma compounds. The components with a higher ROAV value had a greater contribution to the overall flavor of the peach sample.

As can be seen from Table 3, 14 key aroma substances were detected in “Yuanmeng,” among which (E,E)-2, 6-non-adienal (14) and para-cymene (4) had a great influence on its overall flavor. There were three compounds with an ROAV ≥ 1 of “Yihe,” and (E)-β-ionone (24) had a great effect on its flavor. There were 11 kinds of peach in “Yuandong,” and γ-decalactone (15) and linalool (21) had a great influence on its flavor. There were seven species of “Wanhujing,” and γ-decalactone (15) and hexanol (18) had a great influence on its flavor. There were three kinds of “Baifeng,” and the influence of γ-decalactone (15) on its flavor was great. There were seven kinds of “Huangguifei,” and γ-dodecalactone (23) and α-pinene (27) had a great influence on its overall flavor. There were five kinds of “Jinyuan,” and (E,E)-2, 4-hexadienal had a great influence on its overall flavor. “Jinxiu” only had one kind of compound with an ROAV ≥ 1, which was (E)-β-ionone (24). There were nine kinds of key flavor compounds in Ruipan-19, and dihydro-β-ionone (25) had a great influence on its overall flavor. There are eight kinds of “Yulu,” and dihydro-β-ionone (25) and eugenol (39) had a great influence on its overall flavor.

**FIGURE 5:**
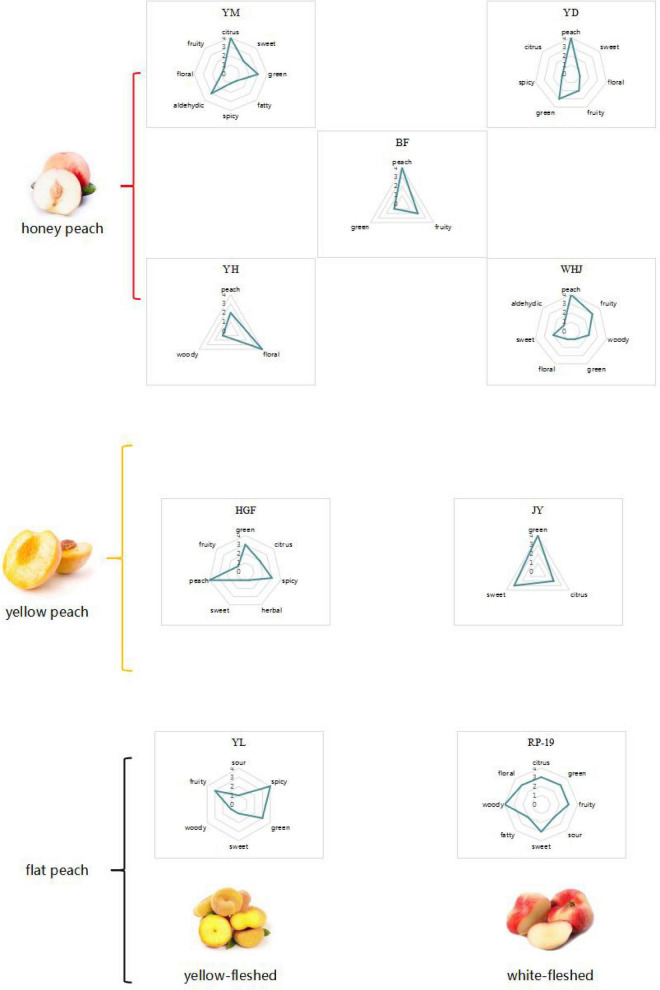
Flavor profile of each peach sample.

According to the volatile components and aroma of ROAV ≥ 1, a radar map was made. As no more than three key aroma substances were detected in “Jinxiu” yellow peach, the map could not be made. According to the analysis of ROAV ≥ 1 components of “Jinxiu” yellow peach, the overall flavor of “Jinxiu” yellow peach was floral fragrance. It can be seen from Figure 5 that the main odor of different peach fruits was citrus flavor. “Huangguifei,” “Yuandong,” “Wanhujing,” and “Baifeng” peach had the main flavor was peach. The main flavor of “Yulu” was spicy, and that of “Ruipan-19” peach was woody.

### Principal component analysis based on gas chromatography–mass spectrometry

**FIGURE 6:**
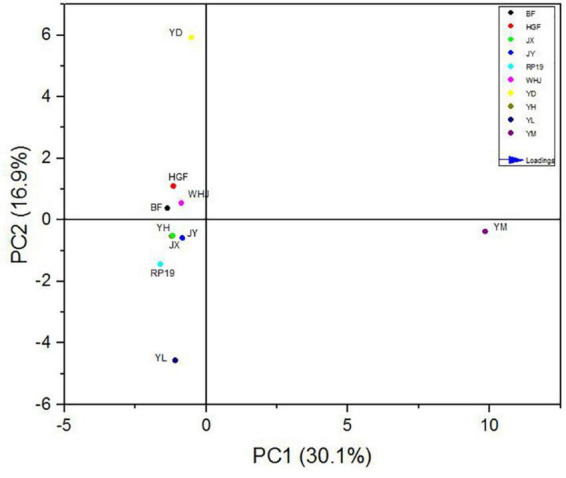
Scatter plot of principal component analysis (PCA) of 10 peach samples.

In order to further study the effect of volatile flavor substances on peach fruit flavor, principal component analysis (PCA) was carried out on the compounds contributing to peach fruit flavor, and Figure 6 is made by the volatile substance with an ROAV ≥ 1 in peach fruit. The accumulative variance contribution rate of PC1 (30.1%) and PC2 (16.9%) was 47.0%. Results are shown in Figure 6, and the chart can be divided into four quadrants, as we can see “Yuandong,” “Baifeng,” “Wanhujing,” and “Huang Guifei” split throughout the second, and “Yihe,” “Jinxiu,” “Jinyuan,” “Yulu,” and “Ruipan-19” split throughout the third quadrant had good aggregation, which meant that the fruit flavor characteristics of these 10 peach samples were similar. “Yuanmeng” was located in the fourth quadrant, which was very different from the other nine kinds, indicating that there were certain differences in the flavor characteristics of different peach fruits.

**FIGURE 7:**
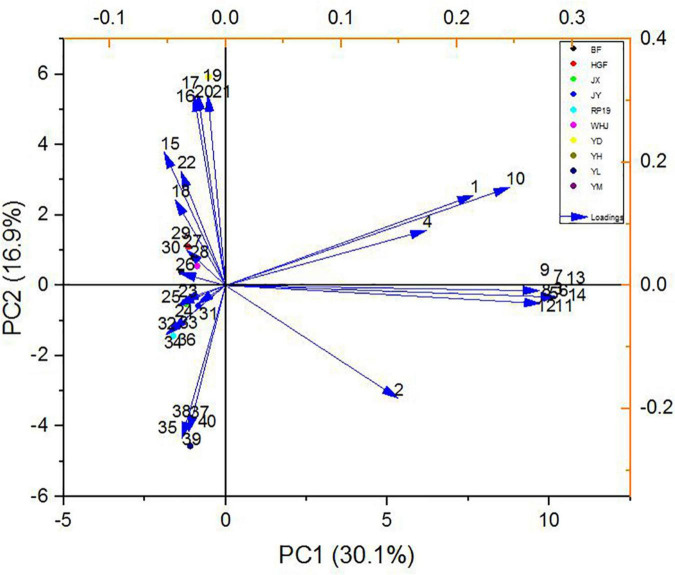
Principal component loading diagram of key flavor compounds in peach samples. The codes correspond to the codes of volatiles in Table 3.

The load values of each principal component represented the reaction degree of the principal component to such substances, which can be used for flavor data analysis, as shown in Figure 7. In PC1, (E,E)-2, 6-non-adienal (14) had the highest positive load and γ-decalactone (15) had the highest negative load, while in PC2, the substance with the highest positive load was β-ionone (17) and the highest negative load was hexyl hexanoate (35). The results showed that (E,E)-2, 6-non-adienal (14), γ-decalactone (15), β-ionone (17), and hexyl hexanoate (35) contributed the most to the overall aroma of peach fruit, which constituted the main aroma of different varieties of peach fruit, and could distinguish different peach fruits well.

In the PCA model, 10 peach samples were clustered into four different groups; their characteristic aroma volatiles were included in each group (Figure 7). “Yuanmeng” clustered in one group with (E)-2-octenal (6), non-anal (9), and (E,E)-2,6-non-adienal (14). “Yulu” clustered with heptanoic acid (38), eugenol (39), and hexanal (40) individually, and also “Yuandong” clustered with β-ionone (17) and linalool (21). The remaining seven peaches “Baifeng,” “Wanhujing,” “Yihe,” “Jinxiu,” “Jinyuan,” “Huangguifei,” and “Ruipan-19” were clustered with γ-dodecalactone (23), (E)-β-ionone (24), dihydro-β-ionone (25), etc.

**FIGURE 8:**
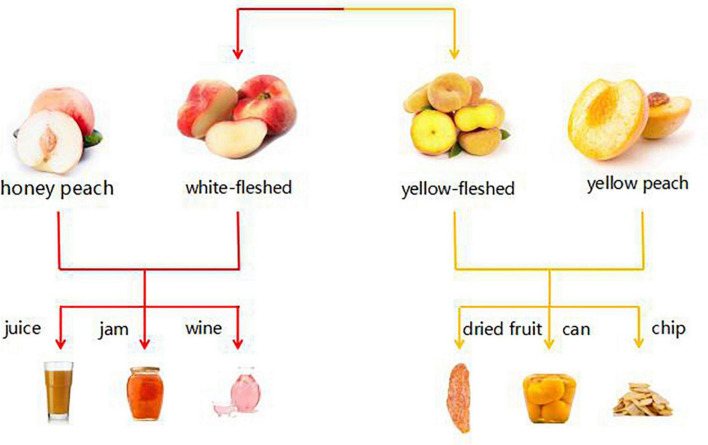
Sketch of possible application of three varieties of peach (honey peach, yellow peach, and flat peach).

According to the preceding classification, we can apply these three varieties of 10 peach samples to various application directions, as depicted in Figure 8. For instance, the aroma of “Yuanmeng” was predominantly sweet, with a trace of aldehyde, and it is a honey peach; therefore, it can be processed into peach by-products such as juice, fruit wine, and jam (26, 27). “Yulu” is a type of flat peach whose aroma characteristics were more reflective of its spicy and fruity aroma, making it an excellent candidate for preserved fruit (6, 28). “Yuandong” is also a honey peach variety, but unlike “Yuanmeng,” its aroma was predominantly green, making it more suitable for dried fruit (29). Among the remaining seven varieties, there were three yellow peach varieties, three honey peach varieties, and one flat peach variety. Despite being of different varieties, their characteristic aroma volatiles were relatively similar, with a predominant peach flavor and a hint of flower. Among the remaining seven varieties, there were three kinds of yellow peach, three kinds of honey peach, and one kind of flat peach. Although they were of different varieties, the characteristic aroma volatiles of them were comparatively similar, mainly peach flavor, with a slight hint of flower. According to Da Rosa Louzada et al. (30); Joshi et al. (31), these peaches were better suited for canning or making wine. If it is not necessary to extend the shelf life of peaches, eating them fresh is the optimal strategy. Honey peaches were more suitable for consumption as juice, jam, etc., or extraction of aroma components for use in daily cosmetics, whereas yellow peaches, due to their hardness and slight green flavor, were more suitable for canned or crispy foods (32–36). But flat peach differs between yellow-fleshed and white-fleshed varieties; if it is white-fleshed, it tends to be similar to the method of honey peach by-products, whereas if it is yellow-fleshed, it tends to be similar to the method of yellow peach.

## Conclusion

In this study, gas chromatography–ion mobility spectrometry was used in conjunction with headspace solid-phase microextraction–gas chromatography–mass spectrometry to analyze and identify the volatile constituents of 10 distinct peach fruit samples. GC-IMS detected a total of 57 volatile compounds, the most volatile of which were esters, which primarily exhibited the aroma characteristics of fruit and flower. The majority of volatile components detected by GC-IMS were small, low-concentration molecules. Although the concentration of these small-molecule organic compounds was low, their molecular weight was small and volatile, making them the easiest to “capture” using sensory or instrumental analysis (37). The composition of volatile components in peach fruits of different kinds can be visualized based on ion migration, despite the fact that the volatiles detected by this method were difficult to be quantified or relatively quantified. The majority of the 88 types of volatile components detected by GC-MS had a high concentration and molecular weight, making them more suitable for headspace solid-phase microextraction technology but insensitive to low-concentration substances. These two technologies enabled the content investigation-related amplification of volatile components in the sample. It demonstrated that the two techniques can be combined to compensate for their respective limitations. Ketones, esters, alcohols, and aldehydes made up a large proportion of various peach fruits as determined by the two techniques. The results of ROAV analysis revealed that the primary flavor substances of 10 peach samples varied. Through principal component analysis of the components, the components with an ROAV ≥ 1 in the aroma components of peach fruits, the cluster analysis of peach fruits revealed that except “Yuanmeng,” the other nine kinds of peach fruits clustered together. The results demonstrated that the volatile substance compositions of “Yuandong,” “Yihe,” “Wanhujing,” “Baifeng,” “Huangguifei,” “Jinxiu,” “Jinyuan,” “Ruipan-19,” and “Yulu” were similar, whereas those of “Yuanmeng” and other varieties were distinct. It was determined that (E,E)-2, 6-non-adienal, γ-decalactone, β-ionone, and hexyl hexanoate contributed the most to the overall aroma of peach fruit and were the primary flavoring substances in three varieties of 10 peach samples, allowing for a good distinction between different peach fruit samples.

Due to its low concentration, GC-MS and GC-IMS are difficult to detect because the relative quantitative method cannot fully explain the composition of volatile flavor compounds in peach fruit and the sulfur compounds in peach fruit, which have a certain effect on the aroma of peach fruit. Therefore, in the next step of this study, the internal standard method and flame photometric detection (FPD) will be used to determine the volatile flavor compounds in peach fruit. Then, aroma recombination and omission experiments are performed to determine the contributions of the selected key aroma compounds. Finally, on the basis of principal component analysis, the partial least squares method (PLS) is applied to analyze the differences between various peach fruits.

## Data availability statement

The original contributions presented in this study are included in the article/supplementary material, further inquiries can be directed to the corresponding authors.

## Author contributions

PS contributed to the conception of the study. YW, XL, CC, and JZ performed the experiment. HJ, XW, and SJS contributed significantly to analysis and manuscript preparation. BX performed the data analyses and wrote the manuscript. TF helped perform the analysis with constructive discussions. All authors contributed to the article and approved the submitted version.
